# Outcome of patients with initial non-mechanical ventilation management in severe pneumonia

**DOI:** 10.1186/2197-425X-3-S1-A95

**Published:** 2015-10-01

**Authors:** A Ortín, R Jiménez, S Rebollo, L Herrera, S Moreno, A Fernández, M Galindo, J Murcia, L Tárraga, A Ojados, OM Téllez, M Contreras, MD Rodríguez, JM Allegue

**Affiliations:** Hospital General Universitario Santa Lucía, Servicio de Medicina Intensiva, Cartagena, Spain

## Introduction

Stablishing when a non-invasive first line approach can be an option in the management of acute respirarory failure due to severe pneumonia represents a major challenge.

## Objectives

To analyze the influence of first line non-invasive respiratory support in the outcome of patients admitted with severe pneumonia.

## Methods

We analyzed retrospective data from patients admitted due to severe pneumonia in our polivalent ICU during a 4-year period. Every patient received antibiotic treatment according to current guidelines.

We collected data related to the specific initial ventilatory therapy, need for therapy escalation and clinical outcomes.

## Results

During the period studied, 113 patients were admitted to our ICU due to severe pneumonia (78 % from community and 22 % from hospital) 36 (31.8 %) were treated initially with invasive ventilation (MV), 45 (39.2%) with non-invasive ventilation (NIV), 5 (4.4%) with high-flow nasal cannula (HFNC) and 27 (23.9%) with conventional oxygen therapy (COT).

NIV was applied to older and more sick (as measured by APACHE II) patients compared to HFNC and COT [63.3 years (CI 95% 58.2-68.5) vs 48.3 (41.5-55) p 0.001; APACHE 17.8 (16.1-19.4) vs 14.9 (12.8-17) p 0.03] ICU stay and mortality was higher in NIV group compared to conventional therapy [8 days (IQR 5-18) vs 4 (2-11) p 0.004; mortality 35.5 % vs 9.1 % p 0.007]

Higher rate of delayed intubation was observed in NIV patients, not reaching statistical difference between groups (40 % vs 24.2 % p 0.15) In those with delayed intubation, duration of MV was higher [13 days (IQR 6-26) vs 8 (3-11) p 0.006] Those patients requiring escalation to mechanical ventilation presented, as was expected, double mortality (50 % vs 11.5 % p 0.001)

Among all patients finally ventilated (63 patients), those with delayed MV had similar age an APACHE II but longer ICU and hospital stay and duration of mechanical ventilation [13 (5-17) vs 19 days (13-39) p 0.005; 18 (12-25) vs 30 days (15.5-47.5) p 0.032; 8 (3-11) vs 13 days (6-26) p 0.006] and a trend to higher mortality (46.4 vs 34.3 %; ns).

## Conclusions

An initial non-invasive oxygen therapy management for severe pneumonia did not impact in outcomes, except for patients finally requiring mechanical ventilation. Non invasive ventilation was probably applied to sicker patients and this fact may explain the worse evolution of this subgroup.
